# Structure and kinetics of indole-3-glycerol phosphate synthase from *Pseudomonas aeruginosa*: Decarboxylation is not essential for indole formation

**DOI:** 10.1074/jbc.RA120.014936

**Published:** 2020-09-14

**Authors:** Annika Söderholm, Matilda S. Newton, Wayne M. Patrick, Maria Selmer

**Affiliations:** 1 Department of Cell and Molecular Biology, Uppsala University, Uppsala, Sweden; 2 Department of Biochemistry, University of Otago, Dunedin, New Zealand; 3 School of Biological Sciences and Centre for Biodiscovery, Victoria University of Wellington, Wellington, New Zealand

**Keywords:** enzyme catalysis, decarboxylase, enzyme structure, enzyme mechanism, enzyme kinetics, biosynthesis, enzyme promiscuity, IGPS, tryptophan biosynthesis

## Abstract

In tryptophan biosynthesis, the reaction catalyzed by the enzyme indole-3-glycerol phosphate synthase (IGPS) starts with a condensation step in which the substrate's carboxylated phenyl group makes a nucleophilic attack to form the pyrrole ring of the indole, followed by a decarboxylation that restores the aromaticity of the phenyl. IGPS from *Pseudomonas aeruginosa* has the highest turnover number of all characterized IGPS enzymes, providing an excellent model system to test the necessity of the decarboxylation step. Since the 1960s, this step has been considered to be mechanistically essential based on studies of the IGPS–phosphoribosylanthranilate isomerase fusion protein from *Escherichia coli*. Here, we present the crystal structure of *P. aeruginosa* IGPS in complex with reduced CdRP, a nonreactive substrate analog, and using a sensitive discontinuous assay, we demonstrate weak promiscuous activity on the decarboxylated substrate 1-(phenylamino)-1-deoxyribulose-5-phosphate, with an ∼1000× lower rate of IGP formation than from the native substrate. We also show that *E. coli* IGPS, at an even lower rate, can produce IGP from decarboxylated substrate. Our structure of *P. aeruginosa* IGPS has eight molecules in the asymmetric unit, of which seven contain ligand and one displays a previously unobserved conformation closer to the reactive state. One of the few nonconserved active-site residues, Phe^201^ in *P. aeruginosa* IGPS, is by mutagenesis demonstrated to be important for the higher turnover of this enzyme on both substrates. Our results demonstrate that despite IGPS's classification as a carboxy-lyase (*i.e.* decarboxylase), decarboxylation is not a completely essential step in its catalysis.

Microorganisms synthesize tryptophan using the products of the *trp* operon. The biosynthetic pathway from the metabolic intermediate, anthranilate, starts with its phosphoribosylation, catalyzed by anthranilate phosphoribosyl transferase (AnPRT; the product of *trpD*). The product is isomerized by phosphoribosylanthranilate isomerase (PRAI; the product of *trpF*). The next step is the formation of the indole moiety, which is catalyzed by indole-3-glycerol phosphate synthase (IGPS; the product of *trpC*). This reaction comprises a ring closure of the substrate 1-(*o*-carboxyphenylamino)-1-deoxyribulose-5-phosphate (CdRP) into the product indole-3-glycerol phosphate (IGP). The reaction involves a decarboxylation that makes it irreversible. Therefore, IGPS belongs to the carboxy-lyase family (EC 4.1.1.48). In the last step of tryptophan biosynthesis, IGP is converted into tryptophan by tryptophan synthase (*trpAB*). In some bacteria, such as *Escherichia coli*, IGPS is expressed and functions as a fusion protein with PRAI. The AnPRT-, PRAI-, and IGPS-catalyzed reactions are shown in Fig. 1*A*.

**Figure 1.**
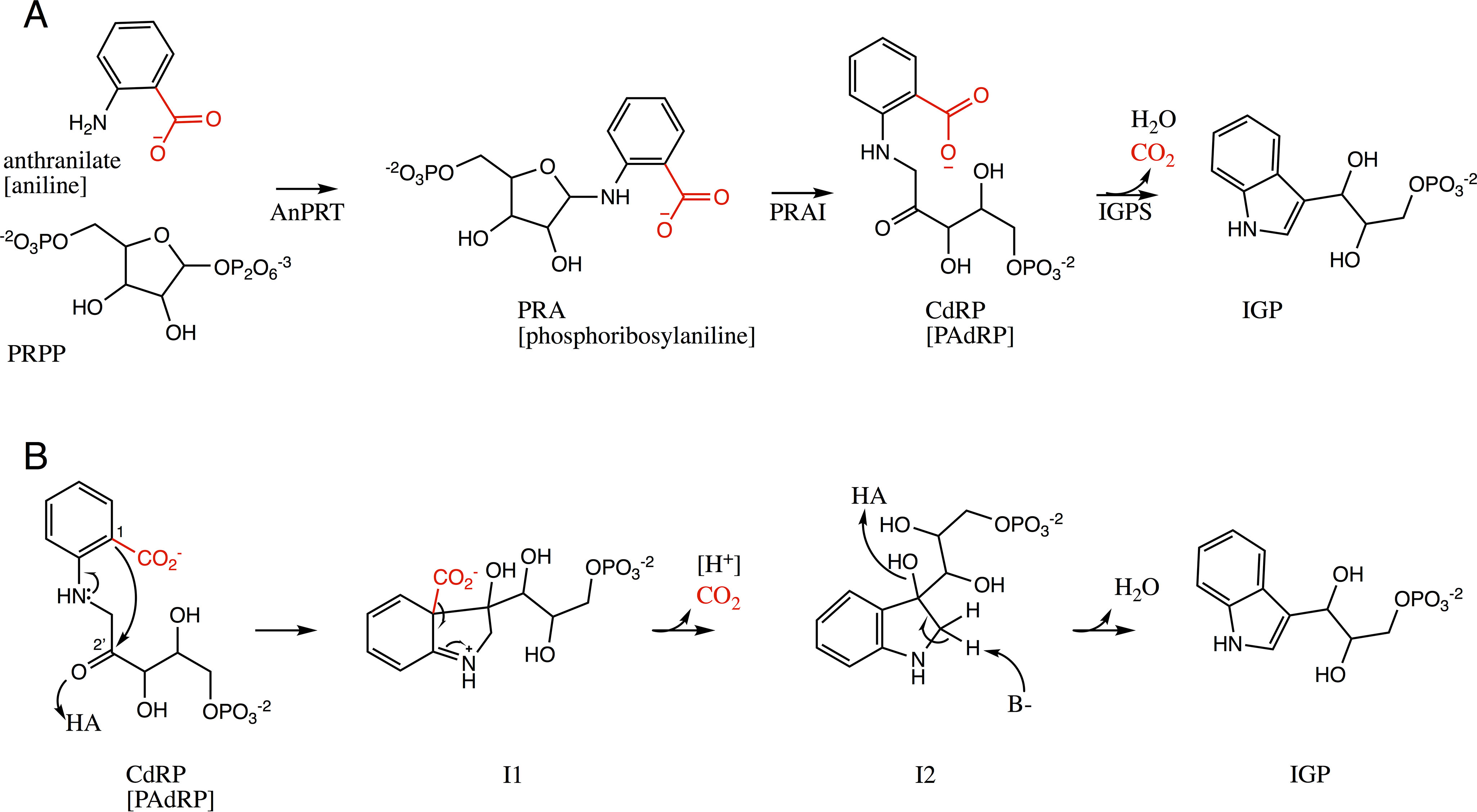
*A*, successive reactions in tryptophan biosynthesis. AnPRT catalyzes the formation of PRA from anthranilate and PRPP. PRAI catalyzes the isomerization of PRA into CdRP. IGPS converts CdRP to IGP via a decarboxylation reaction. The reaction with decarboxylated substrates is equivalent to the *black part*, *i.e.* the carboxylate group (*red*) is substituted by a hydrogen. The names of the decarboxylated substrates are shown in *brackets*: aniline, phosphoribosylaniline, and PAdRP. *B*, reaction mechanism of IGPS (9). The first step is a condensation, where protonation of the ketone by a general acid (HA) results in a carbocation that gets nucleophilically attacked by C1 of the benzyl ring, resulting in intermediate I1. Decarboxylation of I1 makes intermediate I2. Dehydration by protonation of the newly formed hydroxyl by a general acid (HA) and deprotonation of the 1′ carbon by a general base (*B*−) leads to the formation of the indole product. The reaction with the decarboxylated substrate PAdRP is equivalent to the *black part*, *i.e.* the carboxylate group (*red*) is substituted by a hydrogen and the leaving group in step 2 is H^+^.

PRAI and IGPS both adopt the (βα)_8_-barrel fold. In the past two decades, they have emerged as model systems for understanding the evolution of biosynthetic pathways and for the evolvability of the (βα)_8_-barrel (1). IGPS has also been utilized as a starting scaffold for the *de novo* design of entirely new enzymes (2). Because it catalyzes the formation of the indole heterocycle, it is of additional interest for chemical synthesis, because indole derivatives are commonly used within the pharmaceutical, agricultural, and material science industries (3). IGPS has also been suggested as a potential drug target, particularly in *Mycobacterium tuberculosis* (4).

Accordingly, the structure, function, and mechanism of IGPS have been investigated. Several structures of the enzyme have been solved, beginning with the structure of the IGPS–PRAI fusion protein from *E. coli* (5). The IGPS structure has a canonical (βα)_8_-barrel fold with an additional N-terminal helical extension comprising two α-helices, α0 and α00, of which α0 forms a lid over the active site (Fig. 2). Helix α0 is known to be important for substrate binding affinity, because removal of the residues corresponding to helix α0 by trypsin digest in the IGPS enzymes from *Sulfolobus solfataricus* and *Thermotoga maritima* resulted in dramatic increases of *K_m_* (6). Structures of IGPS from the hyperthermophilic archaeon *S. solfataricus* (*Ss*IGPS) in complex with the substrate CdRP, the substrate analog reduced CdRP (rCdRP), and the product IGP have provided important insights into the active-site features (7). Although the deoxyribulose 5-phosphate component of the substrate and product stays fixed during the reaction, the indole ring of IGP binds 5–6 Å away from the anthranilate moiety of CdRP, occupying an adjacent hydrophobic pocket. CdRP and the analog rCdRP adopt extended “unproductive” conformations in which the distance between the carbon atoms is too large (4.5 Å) to be joined in pyrrole formation. Hence, conformational rearrangements are required during the reaction and are supposedly guided by the flexible βα-loop 1, which has been demonstrated to be important for catalysis, as well as binding (8).

**Figure 2.**
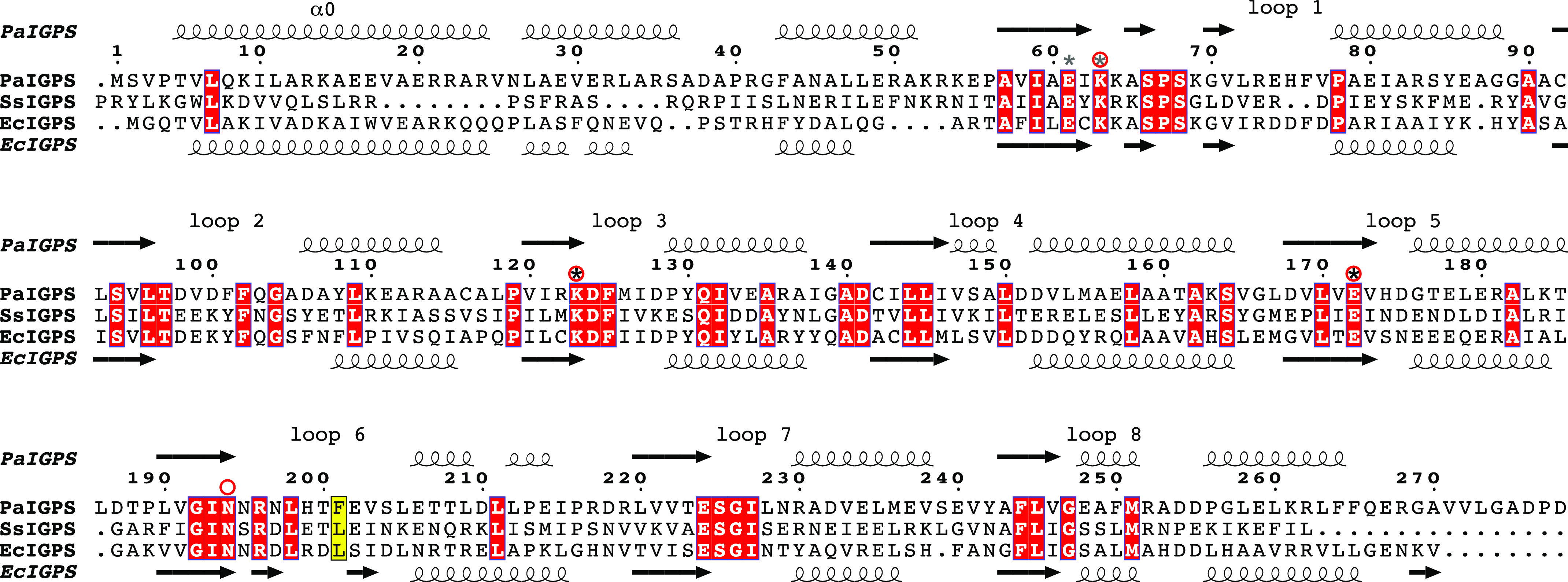
**Structure-based sequence alignment of IGPS from *P. aeruginosa*, *S. solfataricus*, and *E. coli*.** Secondary structure elements are shown for *Pa*IGPS above the alignment and for *Ec*IGPS (PDB entry 1PII (5)) below the alignment. Helix α0 and the βα loops on the catalytic side of the barrel are labeled above the alignment. The suggested catalytic residues (Lys^123^ and Glu^172^) according to Hennig *et al.* (7) are marked with *black asterisks*, and the alternative catalytic residues (Lys^63^ and Glu^61^) suggested by Zaccardi *et al.* (13) are marked with *gray asterisks*. Essential residues (11, 12) are marked with *red rings*. The residue targeted for mutagenesis in this study is marked in a *yellow box*. The figure was prepared with ESPript (31).

Despite numerous kinetics studies, aspects of the IGPS reaction mechanism remain unsettled. For instance, there are discrepancies in the literature regarding the identities of the catalytic residues and the rate-determining step of the reaction. In 1972, Parry (9) proposed a reaction mechanism with three sequential steps of condensation, decarboxylation, and dehydration, involving the intermediates I1 and I2 (Fig. 1*B*), catalyzed by a general acid and a general base. This general mechanism was supported by tandem MS in which a molecular species with a fragmentation pattern agreeing with intermediate I1 was identified (10). The conserved residues Lys^53^, Lys^110^, Glu^159^, and Asn^180^ (*Ss*IGPS numbering) have been demonstrated to be essential for activity (corresponding to Lys^63^, Lys^123^, Glu^172^, and Asn^194^ in *Pa*IGPS, Fig. 2) (11, 12), and based on the *Ss*IGPS-ligand structures, Lys^110^ was suggested to act as catalytic acid for both condensation and dehydration, whereas Glu^159^ was suggested to act as catalytic base in the dehydration step (7). Later, Zaccardi *et al.* (13) argued that it is unlikely for Lys^110^ to act as catalytic acid in both reaction steps because there is no clear mechanism for its reprotonation. They instead suggested Lys^53^ and Glu^51^ to constitute the catalytic acid and base, respectively, for the dehydration step. As support for their hypothesis, they demonstrated shifted pH-rate profiles when either of these residues were mutated.

Aiming to address what is rate-limiting for the reaction, the individual steps have been analyzed by means of (pre-)steady-state kinetics, pH profiling, and solvent kinetic isotope effect (4, 8, 14). At moderate temperatures, the rate-limiting step of the thermophilic *Ss*IGPS was shown to be product release (8, 15), but at 75 °C the reaction appeared to be limited by the proton transfer step during dehydration. In contrast, for the mesophilic *E. coli* enzyme, a chemical step prior to CO_2_ release (*i.e.* condensation) was suggested to be rate-limiting (14). Because the reaction involves removal of CO_2_, Smith and Yanofsky (16) tested whether *E. coli* IGPS (*Ec*IGPS) could turn over the decarboxylated substrate, 1-(phenylamino)-1-deoxyribulose-5-phosphate (PAdRP), to yield the native product, IGP. No such activity was detected, leading to the conclusion that the carboxyl group is essential for IGPS activity. This conclusion has been cited (7), but the mechanistic imperative for decarboxylation has not been addressed in any previous study.

We previously reported the initial characterization of IGPS from the opportunistic Gram-negative pathogen, *Pseudomonas aeruginosa* (17). This enzyme, *Pa*IGPS, has a higher *k*_cat_ value than the other IGPSs that have been characterized (*k*_cat_ = 11 s^−1^). This has made it useful in coupled activity assays of other Trp biosynthetic enzymes, such as evolved HisA mutants with PRAI activity (18). We also showed that deleting *trpC* from *P. aeruginosa* incurred a fitness cost of ∼11% in synthetic cystic fibrosis sputum medium (17), suggesting that inhibition of IGPS may be a useful strategy for helping to clear *P. aeruginosa* infections from the lungs of cystic fibrosis patients, where tryptophan levels can be undetectably low (19).

With our unusually active IGPS in hand, we have sought to revisit the mechanism of the enzyme. During experiments to evaluate substrate specificity in our HisA mutants with PRAI activity (18), we serendipitously discovered that one such mutant, HisA(D10G, dup13-15, G102A, Q24L, V14:2M), had activity with the decarboxylated analog of phosphoribosylanthranilate (20). The product of this reaction is the decarboxylated analog of CdRP, PAdRP (Fig. 1), enabling tests for IGPS activity on decarboxylated substrate. By using a combination of kinetics, structure analysis, and mutagenesis, we here demonstrate that decarboxylation is not completely essential for the mechanism of *Pa*IGPS, although the catalytic efficiency with PAdRP is 41,000-fold reduced compared with CdRP. The structure of *Pa*IGPS also allows us to pinpoint structural differences between homologous IGPS enzymes that are important for substrate ambiguity.

## Results

### Structure of P. aeruginosa IGPS in complex with rCdRP

**Figure 3.**
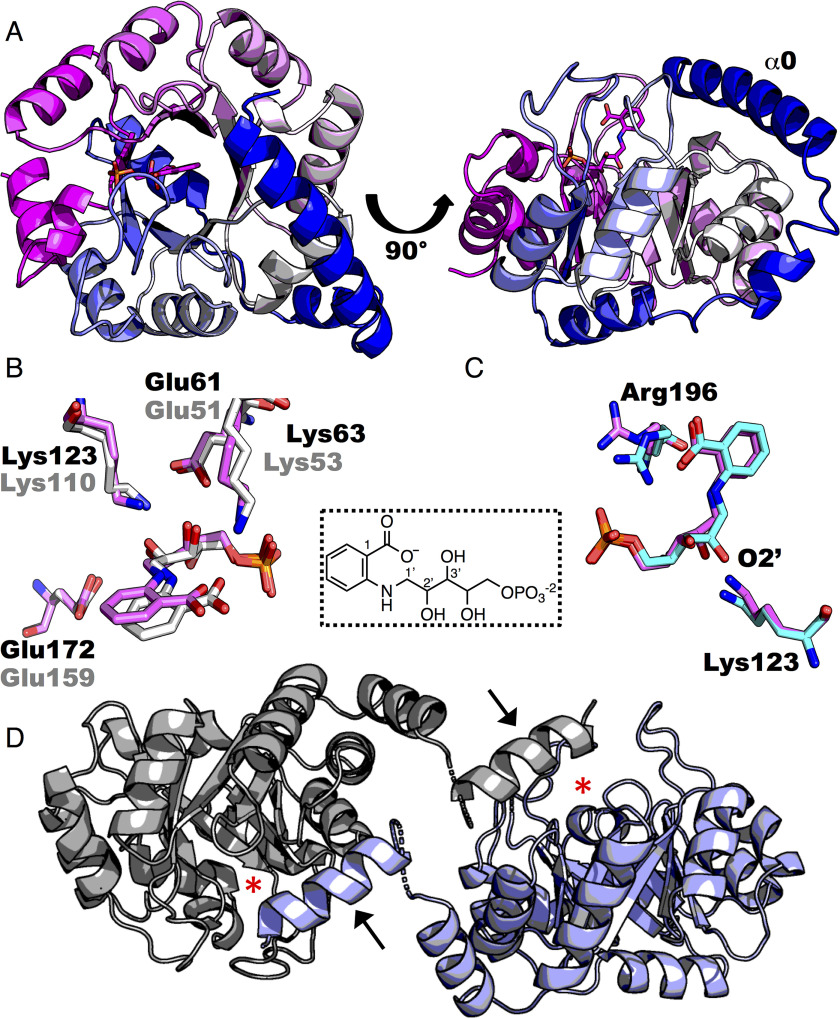
**Structure of *Pa*IGPS–rCdRP.** *A*, structure of *Pa*IGPS chain A in complex with rCdRP, colored in a *blue–white–magenta spectrum* from the N terminus to the C terminus. *B*, suggested catalytic residues in *Pa*IGPS chain A (*purple*, *black text*) and *Ss*IGPS (PDB entry 1LBF) (*white*, *gray text*). *C*, comparison of ligand conformation in *Pa*IGPS chain A (*purple*) and chain B (*cyan*). The chemical structure of the substrate analog rCdRP with atom numbering is shown in the *inset*. *D*, domain swapping of C-terminal helix α0 (marked with *black arrows*) in *Pa*IGPS, chain G. The positions of the active sites are marked by *red asterisks*, and the disordered region between α0 and α00 is *dashed*.

The *Pa*IGPS–rCdRP complex crystallized in space group P2_1_2_1_2 in a large unit cell containing eight enzyme molecules per asymmetric unit. The enzyme was purified as monomer, and its monomeric state in solution was further verified by small-angle X-ray scattering (data not shown). Similar to previous IGPS structures, the enzyme forms a (βα)_8_-barrel with additional N-terminal helices (Fig. 3*A*). Seven of the protein chains (A–F and H) are complete apart from one or two missing residues in the N terminus and ∼20 missing residues in the C terminus, including the vector-derived sequence TSGHHHHHH. In addition, the eighth chain (G) has a partly disordered N terminus and βα-loop 2. The root-mean-square deviation (RMSD) of chains B, C, D, E, F, and H is ∼0.2 Å for 230–250 C_α_, and the RMSD of chain G is 0.3 Å over 213 C_α_ atoms when superimposed on chain A.

The substrate analog, rCdRP, is bound in the active site of the seven more ordered chains. rCdRP is enclosed in the active site by the helix α0 and βα-loops 1, 2, and 6. The suggested catalytic residues (Lys^123^, Glu^172^, Lys^63^, and Glu^61^) adopt similar conformations and identical interactions with the ligand rCdRP as in the *Ss*IGPS structure (Fig. 3*B*). The C2' position of rCdRP is in the R configuration as it is in the *Ss*IGPS complex (7), and the conformation of the ligand is identical in chains A, C, and E. The same conformation is modeled in chains D, F, and H, but there, the electron density is poor in the C1'–C2' region (Fig. S1), indicating flexibility around O2'. In chain B, the same conformation of rCdRP does not fit the electron density, and the ligand instead adopts a conformation where the C2' hydroxyl is shifted 2.2 Å toward the general acid Lys^123^ (Fig. S1 and Fig. 3*C*). The observed movements in the C1'–C2' region in some chains are possibly coupled to the position of the guanidinium group of Arg^196^ on loop 6, which adopts an “inward” conformation and interacts with both the ligand's carboxylate and phosphate group in chains B, F, G, and H (Fig. 3*C*). The catalytic acid Lys^123^ is responsible for protonation of O2' of CdRP (7) (Figs. 1*B* and 3*B*). The distance between the H-bond donor and the acceptor in the corresponding hydrogen bond is very short in chain B, only 2.1 Å, compared with 2.5 Å in the other chains. Moreover, the unbiased omit electron density around C2' in chain B suggests that the ligand possibly adopts a more planar carbonyl-like conformation, mimicking the substrate CdRP (Fig. S1), but interpretation is limited by the resolution of the structure. The C1'–C2'–C3' angle refines to 117° in the B chain, compared with 113° in chains A, C, and E.

Chain G lacks density for rCdRP in the active site. The N-terminal helix is located on a symmetry axis that distorts the density around the α0 hinge, and this part coincides with the corresponding hinge region of a symmetry-related molecule. Electron density for the α0 (lid domain) helix is, however, visible, and a 14-residue stretch of the helix (residues 11–24) has been modeled as poly-Ala because the register of this stretch is uncertain. The helix adopts an open conformation in chain G, where—through domain swapping—it covers the active site of a symmetry-related molecule (Fig. 3*D*). Residues Val^99^–Phe^102^ of βα-loop 2 are disordered, and the βα-loop 1 density is weak, especially for residues Pro^67^–Gly^70^ at the tip of the loop.

### Activity of IGPS with decarboxylated substrate

**Figure 4.**
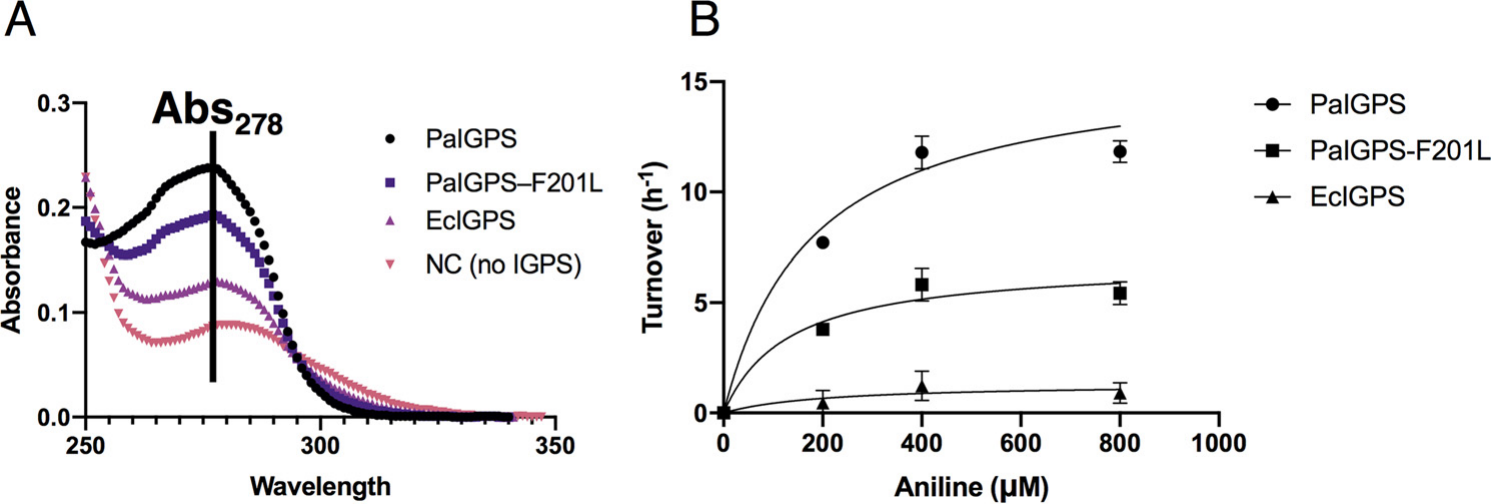
**Activity of IGPS on decarboxylated substrate.** *A*, absorption spectra after 24 h incubation with 2.5 mm *P*aIGPS (*black circles*), *P*aIGPS-F201L (*purple squares*), *E*cIGPS (*magenta triangles*) or without IGPS (*pink triangles*) at 1 mm pathlength. The 280 nm extinction coefficient for EcIGPS is 20400 M^−1^ cm^−1^ and for PaIGPS 5960 M^−1^ cm^−1^, resulting in contributions of ∼0.0051 units for EcIGPS and ∼0.0015 units for PaIGPS to the measured absorbance at this wavelength. *B*, fit of the Michaelis–Menten equation to activity data for *Pa*IGPS, *Pa*IGPS-F201L, and *Ec*IGPS. The figures were made using Prism 8 (GraphPad Software, LLC).

The ability of *Pa*IGPS to turn over the decarboxylated CdRP derivative PAdRP was measured spectrophotometrically by an increase in *A*_278_. The substrate PAdRP was produced enzymatically from aniline and phosphoribosyl pyrophosphate (PRPP) (Fig. 1*A*). Activity with PAdRP was weak but detectable at high substrate concentrations. Product formation was clearly observable when an assay mixture containing 2.5 μm *Pa*IGPS and 400 μm substrate was incubated for 24 h at 25 °C (Fig. 4*A*). The identity of the reaction product, IGP, was verified by high-resolution accurate mass determination using LC-coupled LTQ-Orbitrap MS (data not shown). At 5 μm *Pa*IGPS, the change in *A*_278_ after 24 h corresponded to the production of 380 μm IGP, corresponding to almost complete turnover of the input 400 μm aniline precursor (data not shown). Despite the previous report to the contrary (16), we did also see some turnover of PAdRP by the engineered monomeric variant of *E. coli* IGPS (21) in our highly sensitive assay (Fig. 4*A*).

By sampling the reaction every 30–60 min, we were able to observe a linear increase in product formation over the first 4 h of the incubation. When substrate concentration was varied, the observed reaction rate increased over the range 0–400 μm and then appeared to be saturated at 800 μm. This suggested that the enzyme was obeying Michaelis–Menten kinetics, albeit with very low activity (Fig. 4*B*). Curve-fitting suggested that *Pa*IGPS has a *k*_cat_ of 15.9 ± 1.4 h^−1^ and a *K_m_* of 180 ± 50 μm for PAdRP and *Ec*IGPS has a *k*_cat_ of 1.4 ± 0.7 h^−1^ and a *K_m_* of 200 ± 330 μm for PAdRP.

### Structure-guided mutagenesis

**Figure 5.**
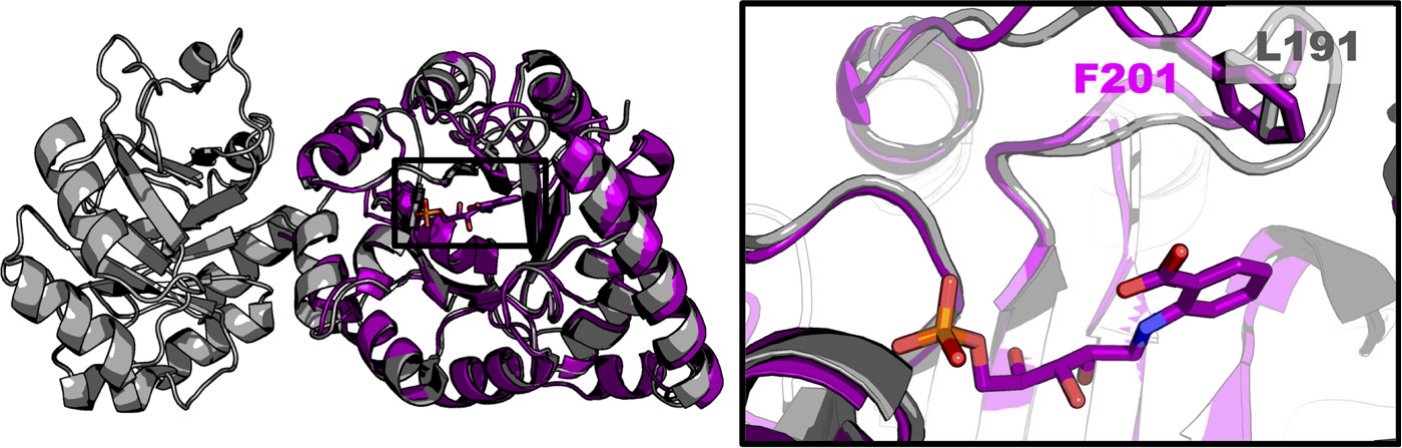
**Superpositioning of *Ec*IGPS–PRAI (PDB entry 1PII, *gray*) on chain A of *Pa*IGPS–rCdRP (*purple*).** The *black box* shows the active site with the ligand rCdRP. Phe^201^ (*Pa*IGPS) and Leu^191^ (*Ec*IGPS) are shown as *sticks*.

The structure of *Pa*IGPS is highly similar to the IGPS domain of the *E. coli* IGPS–PRAI protein (PDB entry 1PII (5)) with an RMSD of 0.9 Å over 201 C_α_ atoms (Fig. 5). Comparison of the active sites shows that most of the residues are conserved. However, in *Pa*IGPS, there is a phenylalanine on active-site βα-loop 6 (Phe^201^), 4 Å away from the anthranilate of rCdRP, whereas in *Ec*IGPS the corresponding residue is a leucine (Fig. 5). We hypothesized that Phe^201^ might improve the ability of *Pa*IGPS to bind the decarboxylated substrate through a π-stacking interaction with its benzyl ring. To test this hypothesis, a *Pa*IGPS-F201L mutant was constructed and tested for activity (Fig. 4*B*). A Michaelis–Menten fit to the data suggested a *k*_cat_ of ∼6.8 ± 0.8 h^−1^ (roughly 40% of WT) and a *K_m_* of 130 ± 60 μm for PAdRP.

### Activity of IGPS with native substrate

To determine how the activities of the IGPS variants with PAdRP compared with the native activity, a similar discontinuous assay was conducted with 400 μm CdRP as the substrate. The activity of *Pa*IGPS under these conditions was determined to 3.0 ± 0.2 s^−1^, which was ∼1000-fold faster than what was measured with PAdRP under the same conditions (11.3 ± 0.7 h^−1^; Fig. 4*B*). On the other hand, the activity of *Pa*IGPS-F201L at 400 μm substrate was 0.42 ± 0.06 s^−1^ with CdRP, which was only ∼300-fold faster than the rate with PAdRP (5.8 ± 0.7 h^−1^; Fig. 4*B*) and similar to the rate of CdRP turnover with *Ec*IGPS (0.29 ± 0.03 s^−1^). The previously reported *k*_cat_ for *Pa*IGPS is 11 s^−1^ (17), and that for monofunctional *Ec*IGPS 3.2 s^−1^ (22).

## Discussion

The tryptophan biosynthesis enzyme IGPS has been heavily studied because of its potential significance for medical and industrial applications. In this work, we have probed the structure and mechanism of IGPS from *P. aeruginosa*, because it has the highest reported turnover number of all IGPS enzymes characterized to date (17).

Our structure of *Pa*IGPS has eight protein molecules in the asymmetric unit showing partly different conformational states of the protein and the ligand rCdRP. The structure of chains A–F and H are overall similar to previous IGPS structures. The active-site residues are generally conserved and form similar interactions with the ligand rCdRP as in the previous structure of *Ss*IGPS with rCdRP (Fig. 3*B*). Lys^123^ is well-positioned to catalyze the proton transfer in the condensation step (Fig. 1*B*). The catalytic residues taking part in the dehydration step are harder to speculate on because of the substantial structural rearrangements taking place during the reaction (7, 8). The product IGP is a closer mimic of the I2 intermediate than rCdRP. Comparing our structure to the *Ss*IGPS in complex with IGP (PDB entry 1A53 (7)) suggests Lys^123^ and Glu^172^ to be more appropriately positioned than Lys^63^ and Glu^61^ to carry out the proton transfers in the dehydration step. The conserved active-site residue Arg^196^ adopts either an inward or outward conformation in the different chains (Fig. 3*C*), which appears to be coupled to the conformation in the C2' region of rCdRP (Fig. 3*C*). Interestingly, the only previously published structure showing the arginine in the inward conformation is the *Ss*IGPS structure in complex with substrate CdRP (Arg^182^ in PDB entry 1LBL (7)), possibly indicating a substrate-activating role of this residue. Mutation of this residue led to a 20-fold higher *K_m_* in *Ss*IGPS (13) but also caused a substantial reduction in the *k*_cat_ of *Ec*IGPS (11).

The least-ordered molecule in the asymmetric unit was chain G, which had three disordered regions (part of helix α0, βα-loop 1, and βα-loop 2). Interestingly, flexibility of these regions is known to be of functional importance. Helix α0 is important for substrate binding (6), and βα-loop 1 motions have been coupled to chemical steps of the reaction, which involves repositioning of the ligand's benzyl ring from one binding pocket to another (8). βα-Loop 2 is suggested to help govern these motions (23). Helix α0 in chain G adopts an open conformation that has not been observed in any previous IGPS structure. However, a different open conformation of helix α0 is observed in the structure of IGPS from *Brucella melitensis* (PDB entry 3TSM). Chain G lacks rCdRP in the active site, emphasizing the role of helix α0 in ligand binding. Moreover, the disorder of these three regions reflect their innate flexibility in the apo state and suggest an induced fit mechanism of substrate binding.

The canonical reaction mechanism (9) proceeds through the sequential steps of condensation, decarboxylation, and dehydration (Fig. 1*B*). Previous studies have focused on the acid–base catalyzed mechanism of condensation and dehydration, without addressing the decarboxylation step. However, requirement of the substrate carboxyl group was demonstrated in an early study of the IGPS–PRAI fusion protein from *E. coli* (16), leading to the conclusion that this group plays an essential role in the mechanism. Here, we have substituted PRAI with a HisA mutant that turns over decarboxylated phosphoribosylanthranilate (PRA) to form the decarboxylated IGPS substrate PAdRP (Fig. 1*A*) and used a more sensitive assay to detect IGP formation from this substrate. For the first time, we have demonstrated that IGPS displays substrate ambiguity toward the decarboxylated substrate PAdRP (Fig. 1*B*). Hence, decarboxylation is not completely essential for the mechanism of IGPS, despite the enzyme belonging to the carboxy-lyase family.

Parenthetically, our coupled assay used the AnPRT from *Acinetobacter baylyi* (24) to produce phosphoribosylaniline (Fig. 1*A*). In addition to its native substrate, anthranilate, this enzyme is able to accept aniline as an alternate substrate. The *M. tuberculosis* AnPRT is known to display other types of substrate ambiguity (25).

The activity of *Pa*IGPS with PAdRP was weak but detectable. The *k*_cat_ at 25 °C was ∼15.9 h^−1^ (0.0044 s^−1^), which is 2,500-fold lower than the reported CdRP turnover at 37 °C (17). The *K_m_* was less severely affected, increasing 17-fold (from 11.3 to 180 μm) with the decarboxylated substrate. Overall, this suggests a catalytic efficiency (*k*_cat_/*K_m_*) of ∼24 s^−1^ m^−1^ for the synthesis of IGP from PAdRP. This is 41,000-fold lower than the corresponding activity toward the native substrate, CdRP (17) (Fig. 1*B*). From our measurements with the native substrate CdRP, we observed that our assay reported lower activities than previously published for *Ec*IGPS and *Pa*IGPS. This is not surprising because our discontinuous assay was optimized for long time measurements to detect low activities. For fast reactions, continuous assays will produce more accurate estimates of initial velocity.

Our study demonstrates that a carboxylated substrate is not essential for activity; however, the difference in activities emphasizes the mechanistic importance of having the carboxylate group. For the condensation step to occur, the conjugated π-bond system of the benzyl has to break (Fig. 1*B*). The carboxylate group will be crucial for activating the C1 carbon for nucleophilic attack. The reaction with decarboxylated substrate—in the absence of the activating group—is chemically very unlikely to occur and would not be possible without an enzyme. Further, the decarboxylation step to yield intermediate 2 (I2) from I1 becomes a deprotonation step when PAdRP is the substrate (Fig. 1*B*). Decarboxylation is not normally rate-limiting (8, 14) and should be thermodynamically favorable because the conjugated ring system is restored, and the liberation of CO_2_ provides an entropic advantage, making the reaction irreversible. On the other hand, deprotonation of I1 (Fig. 1*B*) would also restore the conjugated ring system, but it does not offer the same entropy effect. Moreover, the C1 carbon of the tertiary alkyl in I1 ought to be a very weak acid and not prone to release a proton without facilitation by a strong base. The most likely candidate to fulfill this role with this substrate is Lys^123^, because it is structurally well-positioned to carry out the deprotonation assuming that the product IGP (PDB entry 1A53 (7)) poses a good mimic of the I1 intermediate. In addition, it is deprotonated in the first step of the mechanism and hence ready to accept a proton in the second step. The 2,500-fold reduction in *k*_cat_ that we observed for PAdRP, compared with CdRP, might be influenced by both slower step I (condensation) and step II (decarboxylation or deprotonation).

From our measurements with WT *Pa*IGPS on PAdRP, we observe almost complete turnover after 24 h of incubation. This indicates that IGP formation is irreversible or at least strongly thermodynamically favorable even without the decarboxylation.

We detected a very low level of IGP formation from PAdRP using the *E. coli* enzyme *Ec*IGPS. This was approximately an order of magnitude lower than the activity we quantified for *Pa*IGPS (Fig. 4*B*). By examining the structures of the two enzymes (Fig. 5), we identified only two nonconserved residues in the active site: Glu^248^ in *Pa*IGPS is Ser^237^ in *Ec*IGPS and Phe^201^ in *Pa*IGPS is Leu^191^ in *Ec*IGPS. Glu^248^ is positioned on βα-loop 8 (Fig. 2) and only participates by backbone interaction in phosphate binding, far from the carboxylate group. Phe^201^ is positioned on βα-loop 6 and participates in enclosing the anthranilate side of the ligand (Figs. 2 and 5). We hypothesized that the Leu-to-Phe substitution contributed to the higher ability of *Pa*IGPS to turn over PAdRP. As predicted, introduction of the F201L mutation into *Pa*IGPS reduced its activity with PAdRP. Although *K_m_* remained similar, *k*_cat_ was reduced by ∼60%.

Interestingly, the F201L mutation caused a more dramatic–almost 90%–reduction in activity of *Pa*IGPS with the native substrate CdRP, making it as poor as *Ec*IGPS. Two observations can help to rationalize this. First, Phe^201^ is in close proximity (∼4 Å) to the anthranilate group of rCdRP, but based on superposition of our structure onto *Ss*IGPS in complex with the product IGP (PDB entry 1A53 (7)), it would be ∼9 Å from the indole moiety of the product. This suggests that Phe^201^ can only contribute in the early steps of the reaction. Second, the similar *K_m_* but varying *k*_cat_ for *Pa*IGPS and *Pa*IGPS-F201L with PAdRP suggests that Phe^201^ influences a chemical step of the reaction. Combining these two observations, we can speculate that Phe^201^ helps to guide the condensation step of the reaction and that this is the rate-limiting step of the native reaction, whereas with the decarboxylated substrate, the deprotonation step (from I1 to I2) has become rate-influencing (Fig. 1*B*). In this regard, *Pa*IGPS behaves as the mesophilic enzyme *Ec*IGPS, but not as the thermophilic ortholog *Ss*IGPS (14).

In conclusion, we have solved the structure of IGPS from the opportunistic pathogen *P. aeruginosa*, a possible inhibition target to clear infections in the lungs of cystic fibrosis patients. In the structure we observe partly varying conformation of ligand and enzyme in the different molecules of the asymmetric unit. *Pa*IGPS has the highest reported turnover, and from structure-guided mutagenesis we find that an active-site phenylalanine is responsible for its high activity compared with other IGPS enzymes, possibly by guiding the rate-limiting condensation step. Finally, we could determine promiscuous activity with the decarboxylated analog of the substrate, despite the fact that the carboxylate group was expected to play essential roles in the mechanism of the reaction. Although this activity is low, it opens up the possibility to screen for other forms of substrate ambiguity, for which *Pa*IGPS should be a good candidate because of its high turnover. Further, it shows the strength of enzymes in catalyzing otherwise “nearly impossible” chemical reactions.

## Experimental procedures

### Site-directed mutagenesis

Site-directed mutagenesis of *Pa*IGPS to produce the F201L mutant was carried out according to the QuikChange II protocol. The primers to introduce the mutation into the pLAB101-*PatrpC* plasmid were 5′-caacctgcacaccttagaggtgagcctgg-3′ and 5′-ccaggctcacctctaaggtgtgcaggttg-3′ (mutagenized nucleotides underlined). The mutated plasmid was used to transform *E. coli* Top10 cells. Transformed cells were spread on LB agar plates containing 100 μg/ml ampicillin. Single colonies were used to inoculate cultures, from which plasmid DNA was prepared using QIAprep Miniprep kit (Qiagen). The presence of the desired mutation was confirmed by sequencing.

### Protein expression and purification

All enzymes (*A. baylyi* AnPRT (*Ab*AnPRT), *Salmonella enterica* HisA(D10G, dup13-15, G102A, Q24L, V14:2M), *Pa*IGPS, *Pa*IGPS-F201L, *Ec*IGPS) were expressed in *E. coli* BL21 (DE3) cells from the plasmids pLAB101-*PatrpC* (17), pWP120-*EctrpC*, pCA24N-*AbtrpD* (24), and pEXP5-CT-*SehisA-*DA26438 (18). The cells were grown in LB medium supplemented with 50 μg/ml ampicillin or, in the case of *Ab*AnPRT, 20 μg/ml chloramphenicol. Overnight cultures of 5–10 ml were used to inoculate 1-liter cultures, and expression was induced by the addition of 0.5 mm isopropyl β-d-thiogalactopyranoside when *A*_600_ reached ∼0.5. Expression of IGPS variants and *Ab*AnPRT was conducted at 32 °C for 4–6 h. Expression of the *Se*HisA (D10G, Dup13-15, G102A, Q24L, V14:2M) mutant was conducted at room temperature overnight. The cells were harvested by centrifugation and stored at −20 °C until purification.

For purification, the cells were resuspended in lysis buffer (50 mm Tris-HCl, pH 7.5 or 8, 300 mm NaCl, 5 mm 2-mercaptoethanol), and DNaseI and cOmplete protease inhibitor (Roche) were added to the lysate. The cells were lysed using a cell disruptor (Constant Systems) and centrifuged at 30,000 × *g* for 30–60 min to separate cell debris. The lysate was filtered using a 0.45-μm syringe filter and incubated under slow rotation in a lysis buffer–equilibrated Ni^2+^–Sepharose gravity column for 20–30 min at 4 °C. The column was washed with 70–100 ml of wash buffer (lysis buffer supplemented with 25 mm imidazole), and the His_6_-tagged protein was eluted with elution buffer (lysis buffer with 500 mm imidazole). Protein-containing fractions were pooled and loaded onto a HiLoad 16/60 Superdex 75 column equilibrated with gel filtration buffer: for *Pa*IGPS and the *Se*HisA mutant, 50 mm Tris-HCl, pH 8, 300 mm Na_2_SO_4_, 5 mm 2-mercaptoethanol; and for *Ab*AnPRT and *Ec*IGPS, 50 mm Tris-HCl, pH 8, 300 mm NaCl, 500 and 400 μm MgCl_2_, respectively, and 5 mm 2-mercaptoethanol. The eluted proteins were concentrated using Vivaspin concentrators, flash-frozen in liquid nitrogen, and stored at −80 °C. For crystallization screening, *Pa*IGPS was concentrated to 16 mg/ml. Proteins used for kinetics were concentrated to 5–10 mg/ml. For all proteins, >95% purity was verified using SDS-PAGE.

### Crystallization, data collection, and refinement

Crystallization was done using the sitting drop vapor diffusion method. Prior to setup, *Pa*IGPS was concentrated to 31 mg/ml, into which a small amount of rCdRP (Chemir, EAG) was added as a lyophilized powder. Rectangular cuboid-shaped crystals appeared after several weeks at 8 °C in a drop containing a 1:1 ratio of protein to crystallization buffer (PEG/Ion HT screen (Hampton Research) condition G12, 0.07 m citric acid, 0.03 m Bis-Tris propane, pH 3.4).

The crystal was cryoprotected using reservoir buffer supplemented with 20% glycerol and rCdRP and vitrified in liquid nitrogen. Diffraction data were collected remotely at Beamline I04 at the Diamond Light Source (Didcot, UK) and processed using XDS (26). The structure was solved with Phaser-MR (27) using a truncated (residues 41–264) poly-Ala version of IGPS from *B. melitensis* (PDB entry 3TSM) as the search model. The structure was built using autobuild (28) and manual building in Coot (29) and refined in Phenix.refine (30). The data and refinement statistics are summarized in Table 1.

**Table 1:** **Summary of crystallographic data and refinement statistics**

	*Pa*IGPS–rCdRP
**Data collection**	
Beamline	IO4
Detector	Pilatus 6M-F
Space group	P21 21 2
Unit cell parameters	
*a*, *b*, *c* (Å)	164.7, 150,7, 114.4
α, β, γ (°)	90, 90, 90
Molecules per asymmetric unit	8
Matthew's coefficient (Å^3^/Da)	2.9
Resolution range (Å)	50.0–2.1
*R*_meas_(%)*^a^*	19.1 (82.3)
<*I*/σ(*I*)>*^a^*	7.61 (1.73)
CC_½_ (%)*^a^*	99.4 (72.8)
Completeness (%)*^a^*	100 (100)
Redundancy*^a^*	7.03 (7.01)
**Refinement**	
Resolution range (Å)	47.0–2.1
Reflections/test set	165,894/ 8294
*R*_work_/*R*_free_ (%)	17.3/21.8
No. of atoms	17,566
Protein	16,216
Ligand/ion	221
Water	1129
Average B-factors	
Overall	38.2
Protein	38.0
Ligands	42.0
Solvent	39.3
RMSD from ideal	
Bond lengths (Å)	0.012
Bond angles (°)	1.09
Ramachandran plot	
Preferred (%)	97.7
Allowed (%)	2.1
Outliers (%)	0.2
Protein residues	
Chain A/B/C/D/E/F/G/H	A3–266/B2–266/C2–265/D4–268/E2–266/F2–266/G9–22,*^b^* 25–98, 103–267/H2–265
Ligands	rCdRP, PO_4_, citrate, glycerol

*^a^* The values within parentheses refer to the highest resolution shell.

*^b^* Built as poly-Ala in uncertain register.

### Activity measurements

For activity measurements, stock solutions of 100–150 μm enzyme in reaction buffer with 45% glycerol were made for all enzymes and stored at −20 °C. IGPS activity with decarboxylated substrate was determined using a discontinuous spectrophotometric assay. To produce the decarboxylated IGPS substrate PAdRP, a reaction mixture with the substrates aniline and PRPP and the enzymes *Ab*AnPRT and *Se*HisA(D10G, dup13-15, G102A, Q24L, V14:2M) was incubated overnight at room temperature (16–18 h, 22 °C). The preincubation reaction was set up in reaction buffer (50 mm Tris-HCl, 4 mm MgCl_2_, 0.5 mm TCEP, pH 7.0) with 0, 0.2, 0.4, or 0.8 mm aniline and a 10× molar excess of PRPP (to compensate for spontaneous hydrolysis of the AnPRT product PRA (24)), 5 μm of *Ab*AnPRT, and 4 μm *Se*HisA (D10G, dup13-15, G102A, Q24L, V14:2M). The next morning, the preincubation reaction was aliquoted, and the IGPS reaction was initiated by adding 2.5 μm of either IGPS variant to the different aliquots. For negative control (no IGPS) measurements, the equivalent volume of reaction buffer was added. Each type of reaction was assayed in quadruplicate on two different days using different dilutions of the enzymes, except for the 0 mm substrate and the negative control reactions, which were only done in duplicate. The samples were incubated in darkness at 25 °C for at least 24 h, and UV absorbance spectra between 250 and 350 nm were measured at regular time intervals (every 30–60 min) for the first 5–6 h using a NanoDrop 2000 (Thermo Scientific). Formation of IGP gives a characteristic absorption spectrum with an absorbance maximum at 278 nm. To calculate the reaction rate, the Δ*A*_278_/h from the first 4-h measurements was converted to [IGP]/h using the molar extinction coefficient 5590 m^−1^ cm^−1^ (24). To confirm that IGPS was rate-limiting, a control reaction was conducted with the *Pa*IGPS concentration doubled to 5 μm. The concentration of IGP after 24 h of incubation was determined by the difference in *A*_278_ between time 0 and 24 h divided by the molar extinction coefficient. The kinetics data were fitted to the Michaelis–Menten equation using Prism 8 (GraphPad Software, LLC).

To compare the IGPS activity on decarboxylated substrate with the native activity, the same assay setup was utilized with anthranilate instead of aniline as substrate. For the reactions with anthranilate, the preincubation was done for <5 min, after which 100–400 nm IGPS was added, and the reaction was incubated for 30 min to 2 h. Spectra between 250 and 410 nm (because anthranilate has its absorbance maximum at a higher wavelength than aniline) were measured every 1–2 min for the first 10–20 min. To confirm that the HisA mutant was not limiting for the anthranilate reaction, the identical reaction was also done using 4 μm *Ec*PRAI, but this had no influence on the measured IGPS activity (data not shown).

## Data availability

The structure coordinates have been deposited to the Protein Data Bank with accession code 6Y88. All other data will be shared upon request to the corresponding author (M. S.)

## Supplementary Material

Supporting Information
