# Supplementary material for: Structure and kinetics of indole-3-glycerol phosphate synthase from Pseudomonas aeruginosa: Decarboxylation is not essential for indole formation
Source: J Biol Chem. 2020 Sep 14;295(47):15948–56. doi: 10.1074/jbc.RA120.014936 (PMC7681013; doi:10.1074/jbc.RA120.014936)
Supplement: Supporting Information [file supp_295_47_15948__index.html]

Structure and kinetics of indole-3-glycerol phosphate synthase from Pseudomonas aeruginosa: Decarboxylation is not essential for indole formation — Structure and kinetics of IGPS — Supporting Information 

# Structure and kinetics of indole-3-glycerol phosphate synthase from *Pseudomonas aeruginosa*: Decarboxylation is not essential for indole formation

## Supporting Information

- Supporting Information (to be published online) - Contains supplementary figure 1
