# Supplementary material for: Structure and kinetics of indole-3-glycerol phosphate synthase from Pseudomonas aeruginosa: Decarboxylation is not essential for indole formation
Source: J Biol Chem. 2020 Sep 14;295(47):15948–56. doi: 10.1074/jbc.RA120.014936 (PMC7681013; doi:10.1074/jbc.RA120.014936)
Supplement: Supporting Information [file supp_RA120.014936_161703_2_supp_595866_qghwcq.pdf]

Supplementary material to

Structure and kinetics of indole-3-glycerol phosphate synthase from *Pseudomonas aeruginosa* - decarboxylation is not essential for indole formation

Annika Söderholm[1,4*], Matilda S. Newton[2,5], Wayne M. Patrick[3], Maria Selmer[1*]

[1] Department of Cell and Molecular Biology, Uppsala University, BMC, Box 596, 75124 Uppsala, Sweden

[2] Department of Biochemistry, University of Otago, PO Box 56, Dunedin 9054, New Zealand

[3] School of Biological Sciences and Centre for Biodiscovery, Victoria University of Wellington, Wellington 6012, New Zealand

[4] Present address: Department of Chemistry, Uppsala University, BMC, Box 576, 75123 Uppsala, Sweden

[5] Present address: Department of Molecular, Cellular, and Developmental Biology, University of Colorado Boulder, Boulder, CO 80309, USA and Cooperative Institute for Research in Environmental Sciences, University of Colorado Boulder, Boulder, CO 80309, USA

*to whom correspondence should be addressed:
annika.soderholm@kemi.uu.se or maria.selmer@icm.uu.se

Content:

Supplementary figure 1

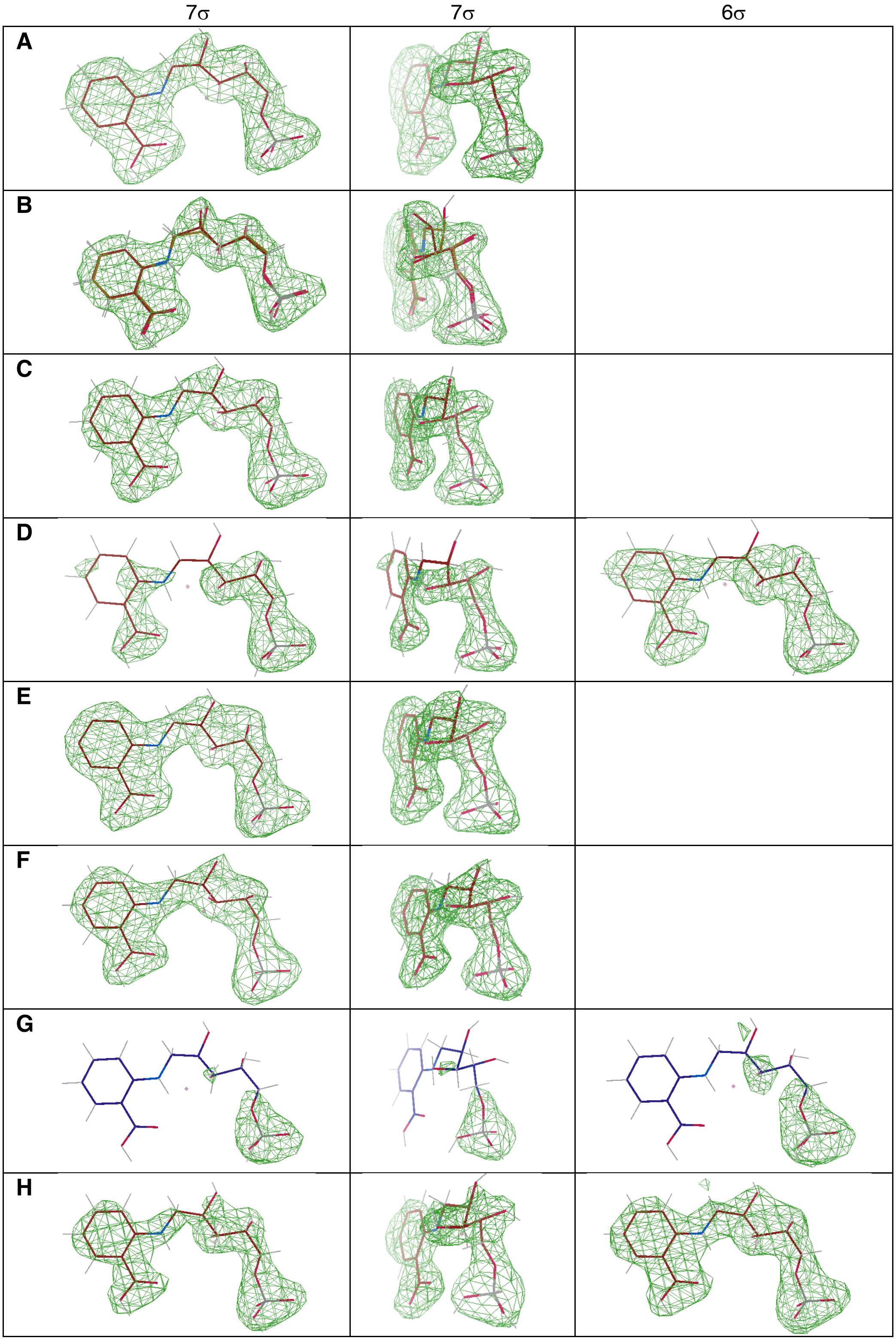

Supplemental figure 1. A-H: Two views of Polder F_o-F_c omit maps (32) contoured at 7σ for the ligand rCdRP in the different protein chains (A-H). Each ligand was omitted individually. Modeled ligands are shown in brown. For chains with weak ligand density (D, G and H), one view is also shown at 6σ. **B:** Modelled conformation in brown, as reference the rCdRP conformation in the A chain is shown in green. The electron density supports a slightly more planar conformation around C2'. **G:** rCdRP as modeled in chain A shown in blue, only phosphate was modeled in this site.
